# Experienced based co design: nursing preceptorship educational programme

**DOI:** 10.1186/s40900-022-00385-3

**Published:** 2022-09-17

**Authors:** Philip Hardie, Aidan Murray, Suzi Jarvis, Catherine Redmond, Co Design Team, Ashley Bough, Louise Bourke, Siobhan Brereton, Andrew Darley, Trish Fahy, Jade Fitzgerald, Liam Fogarty, Brenda Gilmartin, Caoimhe Guilfoyle, John Gilmore, Dawn Hennessy, Julia Kazimierowiska, Lisa Langan, Eoin McEvoy, Joy Melbourne, Claire Murphy, Catrina Ni Dhomhnail, Roisin O’Donovan, Claire O’Sullivan, Jacinta Owens, Maria Slattery, Oana Serb, Grace Walsh, Maureen Whelan

**Affiliations:** 1 School of Nursing, Midwifery and Health Systems, University College Dublin, Dublin, Ireland; 2 Citizen, Dublin, Ireland; 3 Innovation Academy, University College Dublin, Dublin, Ireland

**Keywords:** Experienced based co design, Public and patient involvement, Interpersonal and communication skills training, Nursing preceptorship training, Nursing education

## Abstract

**Background:**

Patients play a central role in nursing preceptorship relationships, a professional educational relationship between a staff nurse and student nurse that is grounded in providing patient care. Yet the patient experiences and perspectives are largely uncaptured in the literature or represented in current preceptorship education programmes. Furthermore, the lack of student, staff nurse & patient involvement in the design of preceptorship education programmes has been noted.

**Objective:**

To use a co-design process to develop an innovative educational programme for developing interpersonal and communication skills among nurses who act as preceptors. We sought to (a) clarify experiences and events from all three members involved in a preceptorship relationship (student nurse, preceptor, and patient (SPP) in order to develop a shared understanding of nursing preceptorship relationships and (b) identify the key informational and educational needs recommended by SPP for the educational programme.

**Methods:**

Using the principles and the iterative process of Experienced Based Co Design (EBCD), data was collected from qualitative interviews and used to inform a series of co-design workshops and the co-production of the new educational programme.

**Results:**

Twenty-six individuals, including undergraduate student nurses, staff nurses, patients, and a team of nursing, educational and educational technologist experts, contributed to developing a blended learning preceptorship educational programme that consists of three core elements (1) six online reusable learning objects, (2) two role play simulations and (3) a virtual reality storytelling simulated experience.

**Conclusions:**

The EBCD process ensured that the educational programme was developed to meet SPP viewpoints associated with fostering positive interpersonal relationships in a nursing preceptorship. EBCD is a valuable framework for developing human-centred educational resources that combine experiential knowledge (experiences) and scientific knowledge (literature-based knowledge). It facilitated the identification and the development of Interpersonal and Communications skills (IP & C skills) training required within a nursing preceptorship relationship, creating an authentic and memorable learning programme. The structure of EBCD harnesses SPP involvement throughout the research and development process, ensuring transparency and continuity of message, scope, and outcomes.

**Supplementary Information:**

The online version contains supplementary material available at 10.1186/s40900-022-00385-3.

## Plain English Summary

Nursing preceptorship is a professional educational relationship between a staff nurse and student nurse based on providing patient care. The preceptor is a role model for the student, demonstrating professional roles and practice responsibilities. Furthermore, the preceptor accesses the student's clinical competence, providing valuable feedback, support and guidance. Therefore, an effective preceptor must develop interpersonal, teaching and competency assessment skills. This paper describes a co-design project that involved engaging nursing students, nurses, former patients, and educational and nursing specialists in creating a new nursing preceptorship education programme. The goal was to develop an educational programme that combined scientific evidence with real-life experiences of nursing students, nurses, and patients focusing on developing nurses' interpersonal and communication skills required to be an effective preceptor. Collaborative co-design workshops were held virtually over several months. Activities included one to one interviews, group discussions and feedback on various drafts of the developing educational programme. Participants provided their stories, opinions, and ideas to create an outline of the new programme. The co-design team then collaborated with educational technologists and a virtual reality production company to complete the state-of-the-art innovative educational programme. The programme is now ready to be piloted. It is hoped this new innovative programme that blends both scientific knowledge with real-life experiences will help nurses develop the interpersonal and communication skills required when interacting with students and patients.

**Supplementary Information:**

The online version contains supplementary material available at 10.1186/s40900-022-00385-3.

## Introduction

Nursing preceptorship plays a central role in nursing education. *Preceptorship* is defined as a professional educational relationship situated within the clinical hospital environment, between a staff nurse (the preceptor) and a graduate or student nurse, involving the delivery of patient care [1]. Therefore, this professional relationship comprises a triadic relationship between the student nurse, preceptor, and the patient (SPP) [2]. The preceptor acts as a role model, supervises, provides guidance and learning experiences, facilitates the socialisation and development of the student in the nursing profession and provides patient care [3, 4]. Furthermore, student assessment and providing continuous ongoing feedback are incorporated within the preceptor role [5]. When students feel their presence is valued and part of the team, and 'preceptors' express empathy towards students, positive interpersonal relationships develop [6, 7]. The quality of the preceptorship relationship can significantly influence the student's integration into the nursing profession and the clinical environment and can affect the student's professional development and delivery of patient care [1, 8].

However, interpersonal attributes demonstrated by preceptors can negatively influence the relationship also, including a hostile cultural environment created by preceptors that imposes a hierarchical status on students [9, 10]. Additionally, preceptors that are dismissive and exclude students from daily activities on the ward result in sometimes hostile or resentful attitudes from students [11, 12]. Furthermore, ineffective feedback methods, i.e., inappropriate locations (In front of the patient) or unconstructive feedback [9, 13], can leave students feeling humiliated and negatively impact their relationship with their preceptor. Indeed, within the researcher's institution, each year, several students report difficulties regarding their lived experiences of preceptorship that occur in the clinical setting. These findings suggest that creating positive interpersonal attributes amongst preceptors is an issue that requires a stronger emphasis in preceptorship education programmes. Despite this a recent scoping review emphasised a lack of focus on interpersonal and communication skills training in current nursing preceptorship programmes internationally[14].

Similarly, positive student-patient relationships are fundamental to the quality of clinical education, delivery of patient care and nursing preceptorships. Patients have valuable perspectives that enrich students' clinical education [15]. However, a recent scoping review [14] highlighted the absence of patients' perspectives on being involved in the preceptorship relationship and the lack of Public and Patient Involvement (PPI) in designing and creating such educational programmes [14]. PPI focuses on the active and meaningful engagement of patients and public members in research processes and activities across a projects cycle [16]. In the UK, the National Institute for Health Research [17] defines PPI as researching with patients and the public, so they are not just participants in the research. Please see Additional file 3 GRIPP2 reporting checklist for PPI involvement in this study. The triadic relations involved in preceptorship include the patient; therefore, this project included patients in the co-design team.

## Methods

This project aims to design a new practical "how to precept" based programme that will allow preceptors to actively engage and reflect on their developing interpersonal and communication skills associated with nursing preceptorship. Studies show that continuous reflection on practice facilitates the development of professional-pedagogical competence and positive interpersonal relations [18]. In addition, the developed educational programme will incorporate crucial skills such as teaching and feedback practices. It will also include the patient's voice and experiences for the first time to provide trainee preceptors with the knowledge and skills to effectively carry out their roles as preceptors and improve interpersonal relations.

Therefore, this study aims to identify key "touchpoints" to determine what SPP liked about their experiences of a nursing preceptorship, what worked well for them in generating positive interpersonal relationships or what caused anxiety or adverse reactions in the professional relationship. It also aims to co-design and co-produce an innovative preceptorship education programme with students, preceptors, patients and experts in nursing and nursing education, incorporating experiential knowledge (touchpoints) and scientific knowledge (literature and expert-based knowledge) applying a user-centred design EBCD approach.

Experienced Based Codesign (EBCD) is a form of participatory action research that involves healthcare professionals and patients/members of the public working collaboratively to develop practical service improvements and patient care improvements in healthcare [19]. It is a multistage process that involves exploring and sharing subjective experiences (narrative-based approach) to enhance healthcare professionals' and organisations' skills and knowledge [20]. In this research project, the team adapted an EBCD process to provide a systematic way to identify and prioritise real-world problems experienced by SPP in a nursing preceptorship to inform the co design and co-production of a new preceptorship educational programme.

Touchpoints play a central role in EBCD. Touchpoints represent critical points with an emotional tone within an individual experience of contact between a service user and any aspect of the service, including interpersonal interactions [21, 22]. Sharing and collaboratively discussing and reflecting on the participant's touchpoints provides an opportunity to see situations from different perspectives and better understand nursing practices [19] or, in the case of this study, nursing preceptorship experiences. Narrative interviewing is a method employed to allow the participant to narrate their experience and have their stories captured [23]. Employing a narrative interview methodology using open-ended questions followed by questions based on the key themes identified in a recent scoping review [14], the researcher aimed to capture the experiences and identify major touchpoints of students, preceptors, and patients (SPP) to later inform the co-design and co-production of the new educational programme.

Twenty-six co-designers were recruited (listed in the acknowledgements section). A small sample size of approximately five participants was present per workshop, in line with expert opinion for effective EBCD workshops [24]*.* Table 1 outlines the diverse sample achieved and the participant demographics. The University Human Research Ethics Committee granted this study's ethics approval.

**Table 1:** Co-design team demographics

Co-design team	Inclusion criteria	Sample	Short description of experience
Student nurses	Undergraduate student nurses in stage 3 or stage 4 of their programme to ensure they had adequate experience of nursing preceptorships	N = 5	Experienced preceptorship across several general hospital sites in the East of Ireland as part of their undergraduate nursing programme. (n = 2) (Year 3), (n = 3)(Year 4)
Qualified nurses that act as preceptors	Preceptors with 5yrs experience or more, precepting undergraduate student nurses	N = 5	Experience ranging from 5-15yrs both nationally (n = 3) and Internationally (Australia, America/(n = 2) as preceptors. Currently working across four different general hospitals throughout Ireland
Members of the public	Members of the public, who were in a general hospital setting in the last two years and were involved in a nursing preceptorship, i.e., teaching session	N = 5	Experienced being a patient in 5 different general hospital sites across Ireland
Experts	Experts were approached to join the co-design team as the project evolved and were chosen based on their extensive knowledge and ability, research, and experience in the required fields	N = 11	Expert knowledge and experience in the fields of nursing education (Lecturer undergraduate and graduate nursing), nursing preceptorship (CPC, Preceptors, Personal Tutor), technology-enhanced education (Educational technologist), pedagogical design (Lecturer/Specialist in the field), universal design (PhD student/Nurse Tutor), storytelling (Expert in the field), simulation, and virtual reality (Expert Clinical Tutor)

The EBCD process facilitated the involvement of all three members of the preceptorship relationship (SPP) in conjunction with Clinical Placement Coordinators (CPCs) (end-users; facilitators of the new educational programme), design experts, nursing experts, educational experts, and researchers at every stage of the design process from problem diagnosis to the design and development of this educational programme and its future implementation. The experiential knowledge SPP brings is essential to helping educators acknowledge multiple realities and meanings of nursing preceptorship relationships. It may also identify aspects of the professional relationship that are poorly understood. The co-design workshops employed a design thinking approach, *a "human-centric"* approach that involves the collaborative generation of ideas, defining and refining issues identified by the SPP. Resulting in iterated and generated solutions through brainstorming and prototype building [25].

Throughout the EBCD process, the researchers ensured authentic involvement from all -co-design team members by implementing Knowles et al.'s framework [26] for authentic co-production. This involved providing a "space to talk", a "space to change", and a "space to talk" again, creating space for shared dialogue and decision making while offering a supportive and friendly environment throughout the project. The project ran for 16 months, commencing in January 2021. Due to Covid-19 government restrictions, the researchers hosted co-design workshops virtually. Table 2 below summaries the steps and outputs using an adapted EBCD approach.

**Table 2:** Steps and outputs using EBCD

Step	Description of step	Outputs
Recruitment of co designers	A purposive sampling approach to recruit a diverse cross-section of the population representing "typical" members of a nursing preceptorship relationship (SPP). Students undertaking a Bachelor of Science (BSc) General nursing degree programme and qualified nurses who held the position of preceptors, were invited to participate via email through a gatekeeper (a senior administrator from the university programme office). Members of the public were recruited through several methods, including social media advertising posts, community advisement boards and word of mouth through the wider community. Recruitment of representation was sought from nursing experts, educational experts, and technology-enhanced educational experts to collaborate with the SPP groups as the project evolved	Recruitment of (n = 26) diverse sample incorporating SPP, nursing, educational and technology-enhanced educational experts from across Ireland. Please see Table 1 and Acknowledgments section.
Semi-structured in-depth interviews	Individual 1–1.5 h interviews with each participant and independently of the group they belonged to (i.e., SP or P) (n = 15) to identify crucial touchpoints for SPP and to Identify educational topics for inclusion in the new programme. Please see Additional file 1 for a complete list of questions and key themes	Detailed qualitative data capturing SPP touchpoints and suggested themes and pedagogy approaches for the new educational programme
Thematic analysis	Dualistic inductive (bottom-up approach analysing SPP touchpoints) and deductive analysis (top-down approach to identify key topics the SPP wished to include in the new educational programme) of the qualitative data gathered. [75]	SPP touchpoints, both positive and negative that influence nursing preceptorship relationships. Key topics the SPP wished to include in the new educational programme
Individual SPP co-design workshops	Each SPP group held a collaborative workshop separately to discuss findings from thematic analysis to reach a consensus on touchpoints and concepts, and teaching methods for inclusion in the programme. The patient group did not wish to have a group workshop; therefore, the lead researcher adapted and encouraged participation by emailing or posting the thematic analysis results from the one-to-one interviews and spoke with each person individually over the phone. The lead researcher then combined all their ideas and populated their chosen outcomes. These were then emailed or posted to all patients for further review and the opportunity to comment	Agreement on touchpoints to represent each group. Development of audio trigger videos. Agreement on key concepts and pedagogical approaches to include in the educational programme
SPP joint co-design workshop	Multi-group design workshop to bring all groups together to work collaboratively. Each SPP's touchpoints were introduced using audio clips. The lead researcher presented a table summarising the educational topics and pedagogical approaches suggested by each group	Agreement on SPP touchpoints to embed in the new educational programme. Agreement on educational themes and pedagogical approaches (PleasesSee Additional file 2)
Specialists co-design workshop	To ensure the new educational programme was also guided by nursing, educational and technology enhanced educational experts, thus grounded in nursing and educational theory and SPP experiential knowledge. An overview of the project, including suggested educational topics and pedagogical approaches, was presented at a virtual workshop, followed by audio presentations of SPP touchpoints. The specialist group discussed and agreed on the proposed outline for the new educational programme, agreeing it would meet its intended outcome to create an authentic learner-centred educational programme for developing interpersonal and communication skills amongst preceptors	Agreement on outputs from SPP joint co design workshop. Agreement to underpin the new programme in behavioural change theory to ensure effectiveness and uptake of the new programme
Presentation of an outline of the new programme	All codesign team members were invited to a presentation of the outline of the new educational programme, offering further opportunities to provide input and feedback. An overview of the suggested timeline for the development of the programme was presented, and a collective agreement was made that the lead researcher would send the codesign team bi-monthly updates on the status of the project	The programme outline was finalised and agreed upon
Codesign/production of RLOS, role play simulations and virtual reality experience	An iterative design process grounded in educational design was implemented to codesign and produce the new educational programme	Prototype of the new educational programme
Celebratory event	The final product was available for all to experience. The lead researcher gave a short talk summarising the project, how it is hoped it will impact nursing education and plans for future research projects investigating the impact of the educational programme on nurses' interpersonal and communication skills and thanked everyone involved in the project	Informal celebration event held on a university campus
Pilot launch of the new programme	This blended learning programme will be piloted across several general nursing hospitals in Ireland that run preceptorship programmes in late 2022. Following implementation, the researchers will seek feedback to establish possible areas for improvement and the perceived impact on learners' future practice regarding their interpersonal and communication skills and their role as a preceptor	Set to be piloted in late 2022

## Results

This study set out to identify critical touchpoints, i.e., personal or crucial memories that shape experiences of nursing preceptorship relationships, to identify key experiential knowledge that later informed the co-design of the new educational programme. Interestingly several similar touchpoints influence the experience and interpersonal relationships from all SPP perspectives, including first impressions, effective communication, and feedback. Other themes included psychological safety and attitude to teaching and assessing students. For full details on SPP Touchpoints and overarching target behaviours to include in the educational programme to address the SPP touchpoints (please see Additional file 2).

Secondly, this project set out to co-design and co-produce an innovative preceptorship education programme incorporating experiential knowledge (touchpoints) and scientific knowledge (literature and expert-based knowledge). It was collectively agreed that the new educational programme would adopt an active blended learning approach consisting of a series of online reusable learning objects (RLOs), face-to-face role-plays and a state-of-the-art virtual reality storytelling experience (Table 3). Two pilot studies previously led by the lead researcher influenced the blended learning programme, having identified that students felt prepared to engage in face-to-face simulation following exposure to RLOs [27]. In addition, his experience of VR storytelling offered the unique ability to provide an immersive storytelling learning experience to supplement learning [28]. RLOs are "a digital resource that can be reused to facilitate learning" [29]. It was collectively agreed that a series of RLOs would be created to promote flexible and autonomous learning online to support a proposed half-day face-to-face simulation. Six RLOs were developed, each addressing a specified learning objective (Table 3).

**Table 3:** Outline of active blended learning preceptorship educational programme

Programme component	Design	Description	Implementation
Online learning resources	Six-month process involved the codesign team following a participatory approach based on the ASPIRE framework (Aims, Storyboarding, Population, Implementation, Release, and Evaluation) [30]. Each RLO unit was peer-reviewed by all codesign team members using the learning object review instrument (LORI) [31], reviewing the overall design, usability, motivation, learning goal alignment, and quality of the content before production began. The content for the RLOs was created, including videos, images, voiceovers, and infographic slides. An educational technologist (A.B.) populated all media on an online platform, Articulate 360 (https://articulate.com))	A series of six online reusable learning objectives (RLOs) was created incorporating a mixture of presenting information, touchpoints, video demonstrations, case study scenarios, interactive exercises, reflective exercises and on the spot feedback Unit 1: Introduction to Interpersonal and Communication skills in Nursing Preceptorship Unit 2: Psychological Safety: Creating a Safe Learning Environment Unit 3: First Impressions & Orientation Unit 4: Teaching in the Clinical Environment Unit 5: Feedback Unit 6: Conflict Resolution	All learning units will be opened up to learners (hosted on Articulate) to complete at their own pace over the three weeks prior to the half-day face to face teaching described below. Each unit takes approximately 40–60 min to complete. Learners can complete the units repeatedly if they wish to do so. An accompanying workbook will also be provided for learners to use with the RLOs to make notes and complete their reflective exercises
Role play simulations	The lead researcher and an academic with a specialist background in simulation-based education and patient safety led the design of the role-play simulations, which was iteratively peer-reviewed by the remaining co-designers. Applying the International Nursing Association for Clinical Simulation and Learning (INACSL) framework [32], the codesign team considered design criteria such as creating a simulation experience that can achieve measurable objectives, is participant-centred and incorporates a level of fidelity that creates a perception of realism to the simulated scenario. The INACSL framework also guided the inclusion of a structured pre-brief session and a structured debrief session following the role plays	Two role-play simulations were designed to provide a safe learning environment to apply the new knowledge and skills from the RLOs Role-play scenario 1: focuses on the learners' ability to provide a psychologically safe learning environment for students Role-play scenario 2 focuses on the learners' ability to provide effective feedback and conflict resolution skills	Learners will be provided with a pre-brief pack that will outline the role play goals and objectives. Learners will be divided into groups of three and alternate between the student, nurse, or patient positions. Each will run for 20 min, followed by 20 min debrief session each time. The learner playing the part of the patient will be provided with an observational feedback sheet to provide structured feedback during the debriefing session
Virtual reality simulation	The lead researcher and a member of the codesign team with a specialist background in storytelling and film production led the design of the VR simulation in collaboration with a VR production company [33]. It was also iteratively peer-reviewed by the remaining co-designers applying the INASCL framework [32] Using critical touchpoints from the interviews and the three-act structure to storytelling (Introduction; rising action; falling action (conclusion)) to structure the storyline, a short 10 min VR storytelling was scripted, storyboarded and peer-reviewed. Using a state of the art 360 VR camera, the simulation was filmed in a simulated hospital ward environment using actors	A state-of-the-art VR simulation was designed to experience nursing preceptorship from a patient's perspective, permitting the learners to "walk in someone else's shoes". The VR simulation depicts a patient's interpersonal interactions with a nurse and student and what it feels like from their perspective, e.g., if a nurse doesn't make eye contact when speaking to the patient or a student who talks over a patient	Leaners will be provided with a pre-brief pack that will outline the VR simulation goals and objectives. Each learner will be provided with a VR headset to use with their smartphones. The VR simulation will run for 7 min. A structured 20 min debrief session will follow the VR experience

Role Play Simulations will allow learners to practice the theoretical and practical information provided in the RLOs. It facilitates the practice of interpersonal and communication skills in a realistic yet safe learning environment [34]. Two role-play scenarios simulating fundamental interpersonal interactions in a nursing preceptorship requiring dynamic and practical interpersonal and communication skills were designed (Table 3).

In addition to the RLOs and role-play simulations, it was decided to develop a new state-of-the-art immersive storytelling experience. This pedagogy combines the emerging world of VR technology with the art form of classical storytelling. During the VR storytelling experience, the learner embodies the role of the patient, feeling the same sensation toward a virtual body within an immersive virtual environment as the biological body in the real world would experience [35]. This will permit trainee preceptors to step into a patient's shoes, experiencing the interpersonal dynamics of a preceptorship relationship from the patient perspective, creating a meaningful and memorable learning experience. It is planned that preceptors will engage with this experience as part of their face-to-face learning, followed by a structured debrief session.

## Theoretical underpinnings of new educational programme

A constructivist approach was applied to the new educational programme, believing that active learners in their learning journey create new knowledge from experiences [36], Kolb's [37] experiential learning theory principles also guide the programme's design. In addition, the principles of Universal Design for Learning (UDL), an inclusive approach, were embedded in the programme to ensure that the programme offered all learners, including those with disabilities or required accommodations, providing equal opportunities to learn [38, 39]. Table 4 outlines Kolb's Experiential Learning Theory & UDL principles strategies for the Nursing Preceptorship Programme.

**Table 4:** Kolb's experiential learning theory and UDL principles strategies for nursing preceptorship programme

Kolb's experiential learning theory	Strategies for blended learning preceptorship programme	UDL principle	Strategies for blended learning Preceptorship programme
Concrete experience	The learner is provided with an opportunity to gain new learning experiences, i.e., online learning resources, role-play simulations and VR simulation	Multiple means of engagement	Identify clear learning objectives. Provide learners with multiple opportunities to achieve the programme's learning outcomes, e.g., RLOs (Articulate), Reflective exercises, Role-Play Simulations, Virtual Reality Storytelling, and debriefing sessions. Provide learner choice
Reflective observation	The learner is provided with an opportunity to reflect through written reflective exercises, self-reported questionnaires, and during simulation debriefing sessions	Multiple means of representation	Use multiple media including audio, visual and text e.g. short video demonstrations. Ensure learners have access to digital documents, e.g., transcript of all RLOs and videos that the learners can edit (colour, font size). Furthermore, all videos have closed captions
Abstract conceptualisation	The learner is provided with an opportunity to learn from the experiences offered in the programme	Multiple Means of Action & Expression	Facilitate active learning. Encourage learner metacognition. Learners can work at their own pace and demonstrate their knowledge in various ways, i.e., in group discussions and role-play simulations
Active experimentation	The learner is provided with an opportunity to plan and try out what they have learned during the half-day simulation training and future preceptorship practice		

Lastly, to improve the effectiveness and uptake of the new educational programme, the expert group suggested that the programme should also be underpinned by behavioural change theory. Identifying the behavioural barriers and enablers to facilitating positive interpersonal relationships would strengthen the likelihood that the new educational programme would act as a catalyst to change practices among established preceptors. Members of the expert group worked with the lead researcher to achieve this. Table 5 outlines the combined approach of behavioural change theory and EBCD and Kurt Lewin's Model of Change Principles and how they were applied to the new educational programme to promote change in preceptorship practice.

**Table 5:** Combined use of EBCD, behavioural change theory and Kurt Lewin's model of change principles

Step of behavioural change theory	How EBCD was utilised	How the new educational programme aims to facilitate change in preceptorship practice	Kurt Lewin's model of change principles	How the new educational programme aims to facilitate change in preceptorship practice
Identify the key determinants of behaviour	A comprehensive review of the literature was completed Priorities for improvement and enablers were identified from the co-design team's touchpoints considered in terms of attitudes and behaviour Expert opinions from the field of nursing were also sought	Key enablers and barriers to facilitating positive interpersonal relations in a nursing preceptorship are embedded throughout the programme, providing the learner with real-life stories, interpersonal skills theory, and practical skills to develop effective IP & C skills for preceptorship practice	The unfreezing stage refers to persuading others that the status quo is not beneficial and encouraging others to view a problem with a fresh perspective, including the patient experience in nursing preceptorship	This programme highlights that the current literature regarding nursing preceptorship primarily excludes the patient experience and perspective, which we argue is incorrect and non-inclusive, as the patient is central to the relationship. Secondly, the programme aims to highlight the importance and negative impact ineffective interpersonal and communication skills can have on the patient experience and the student's learning experience to spark motivation for change among the learners of the educational programme
Identify the techniques that target these determinants	A comprehensive review of educational theory and pedagogical approaches in preceptorship education so participants could select educational and nursing interventions (From validated methods identified in the literature) that they felt would be most effective in the given context and based on their personal experiences	Active learner-centred pedagogy is embedded throughout the programme to facilitate the development of practical IP & C skills associated with a nursing preceptorship	The moving phase refers to the stage in which the researchers will roll out the new educational programme and work collaboratively with CPCs (typically facilitate preceptorship education in hospital settings) to ensure they feel supported in implementing the programme	To enhance the capability, opportunity and motivation for the new programme, the researchers will train the trainer days and ensure all materials for the programme are readily available, including access to virtual reality technology required to roll out the programme successfully. Support will be offered throughout the first run of the programme
Model to fit the target population, culture and context	The design of the programme content closely involved those that will deliver (CPC staff) and receive the educational programme (preceptors) to elicit perspectives on the programme's acceptability, practicality, and cost-effectiveness	The new programme is designed to run as a blended learning programme with a half-day face-to-face simulation training (tackles the issue of releasing staff to attend and costs associated and increases accessibility to the programme)	The refreeze stage refers to when the programme will have been implemented multiple times over two years	The programme aims for a new change in perspective to include the patient in a nursing preceptorship relationship and improve the knowledge and practice of practical interpersonal and communication skills required in a nursing preceptorship. It is hoped that nurses who complete the programme will be a driving force for change in practice

## Discussion

Several papers have discussed nurse preceptors learning needs from a preceptor's point of view [40, 41], while Tsai et al. [42] collected data from both preceptors and student nurses on their preceptorship experiences. However, there is no evidence to date investigating patients' experience of a nursing preceptorship to the best of the author's knowledge. Thus, this empirical research implementing an EBCD process to identify preceptor training needs from all three members of the nursing preceptorship relationship is a unique and novel approach to designing and creating a preceptorship education programme.

In keeping with previous research, this study found that first impressions and a welcoming and open disposition during the student's orientation period strongly influence the basis and ongoing premise of the professional relationship [43–46]. Similarly, patients in this study described the importance of being made feel welcome by a preceptor/student with an open and welcoming disposition as a critical touchpoint that positively influenced their experience by reducing anxiety and creating a feeling of self-worth. These findings align with Holst et al. [47], who reported that patient satisfaction emerged when preceptors made an excellent first impression, were polite and made eye contact. Furthermore, a nurse's body language and tone of voice are strong indicators of how the relationship will evolve at the initial meeting [48]. This study, therefore, further supports the importance of first impressions, including language, tone, and body language, suggesting it plays a vital role in a preceptor creating a positive interpersonal relationship and should be included in preceptorship education programmes.

Including patient in the co-design of the new educational programme identified unique results. The patient co-designers strongly emphasised person-centred communication as an influential touchpoint, citing its importance in facilitating a better patient experience and patient satisfaction. Previous research on patients' healthcare experiences has noted the same [49–52], reporting effective communication and high-quality patient information can result in better patient outcomes and patient satisfaction. In comparison, several studies have also found a correlation between poor nurse-patient communication and patient dissatisfaction [53–55]. Thus, the inclusion of practical interpersonal and communication skills to facilitate person-centred communication in a nursing preceptorship should be included as part of preceptors' education,

Furthermore, patient co-designers indicated the importance of seeking permission prior to a student's learning experience and engaging in student feedback. These results mirror those highlighted in Suikkala et al. [56] scoping review examining patients' involvement in nursing students' clinical education, in which some patients perceived themselves as active participants who facilitated students' learning by sharing knowledge and experience about their care and wellbeing as well as assessed students' performance by providing encouraging feedback. Therefore, it seems patients appreciate the opportunity to contribute to a student's learning process and provide feedback. However, permission should be sought prior to the student being present. Including these views and guidelines to including patients in teaching sessions and feedback in preceptorship education is therefore essential.

Creating bidirectional feedback opportunities was a crucial touchpoint for preceptors in this study. Previous studies also demonstrated an association between preceptor feedback and formulating an open and trusting interpersonal relationship with students [57, 58]. Preceptors in this study also iterated a link between student assessment/feedback and feelings of guilt and pressure when failing a student. Previous studies have demonstrated similar results, with preceptors reporting a reluctance to fail students and the emotional sense of guilt that goes with failing a student [51, 59]. Central to the process of failing a student is providing constructive feedback, yet several studies have highlighted that preceptors feel they need further education in the area of providing feedback to nursing students [51, 60]. Therefore, including practical feedback principles in the new educational programme was imperative to enhance the preceptor's understanding and skills in providing effective feedback.

The co-design team from this study established that a blended learning approach best suited the new preceptorship programme. Many preceptorship programmes are implementing a blended learning approach [20, 61–63] as it reduces the time required away from the ward and increases accessibility and convenience as it permits preceptors to complete a proportion of the programme in their own time [64].

In line with the literature [20, 27, 61, 63, 65] the co-design team selected the inclusion of online learning units as an active pedagogy to prepare preceptors for the half-day face-to-face simulation day of this educational programme. Cited benefits of online learning units (also referred to as reusable learning objects (RLOs) include their ability to present theoretical content interactively and promote autonomous learning that can provide on-the-spot feedback [66].

Similar to the current preceptorship programmes [67–69] this study identified role-play simulations as a practical pedagogy to develop preceptors interpersonal and communication skills.

Currently, there is no evidence of the application of VR storytelling in preceptorship education programmes. However, reported benefits of VR Storytelling in nursing education include learners finding VR more engaging than traditional teaching methods and allowing a 'safe' environment to practise skills without fear of making mistakes [28]. Learners have also reported its suitability for various learning styles and its more flexible mode of teaching [70]. It is hoped that the inclusion of this new innovative virtual reality storytelling experience will provide preceptors with an engaging and authentic learning experience. It will help foster greater empathy for patients involved in nursing preceptorships by walking in their shoes during the VR simulation and thus develop practical and effective interpersonal and communication skills associated with a nursing preceptorship.

To the best of researchers' knowledge, there are currently no published research papers examining the application of EBCD to co-design and co-produce an educational intervention within the context of nursing education. Therefore, this study adds unique insight into implementing an EBCD method to develop an educational intervention. In line with previous EBCD facilitators [71], the researcher found that individual interviews at the beginning of the project engaged the SPP co-designers and enhanced their commitment to the EBCD process. This study confirms that touchpoints are key to unlocking everyday experiences [72] and sparked a highly collaborative process amongst the co-design team when shared [21, 22]. Per the present results, previous studies have demonstrated that collectively reflecting on each group's touchpoints provides a real opportunity to see a situation from different perspectives and better understand practices [19, 73]. This project's approach to embedding the touchpoint narratives throughout the new educational programme signifies an innovative approach to utilising touchpoint narratives beyond the initial design phase of an EBCD intervention and creating an authentic educational programme.

This study suggests that EBCD can be adapted and implemented in a nursing education context and that EBCD can play a critical role in improving preceptorship education programmes by gathering the perceptions and views of SPP to then collaborate with nursing and educational experts to co-design and co-produce the new educational programme. Including SPP and other key stakeholders (CPC, Nurse educators) as co-designers throughout the EBCD process has proved advantageous; it has meant they were key drivers in the educational content of the new educational programme to ensure it spoke to real-world experiences of nursing preceptorships and practical implications to facilitating a preceptorship education programme.

This new education programme addresses some of the current limitations of preceptorship education programmes observed in the literature. Most notably, the inclusion of the patient voice and perspective throughout the programme. Furthermore, it addresses a key concern for both students and preceptors to incorporate foundational interpersonal skills such as creating positive first impressions or language to use when providing feedback that appears to be lacking in many preceptorship education programmes, including the new national mandatory online preceptorship programme, "Preceptorship in Practice" HSEland [74]. A primary focus of the HSE programme is the preceptor's role in assessing student competency and its associated documentation. Limited focus is placed on the practical skills required to facilitate a positive clinical learning experience, including practical interpersonal and communication skills associated with teaching and feedback, and the patient is not mentioned throughout the programme. Furthermore, this programme is online; therefore, preceptors are not offered the opportunity to apply new knowledge gained from completing the programme in a safe environment, such as role-play simulations included in this new programme. The preceptorship education programme designed in this study was recently approved as a Category 1 (CEU 8 points) by NMBI until Jun 2024. Therefore, it is hoped this new educational programme can support ongoing professional development and be implemented as a preceptorship update course, which NMBI recommend every 2–3 years for all preceptors [5]. It will benefit practising preceptors to reflect on their practice and develop their interpersonal and communication skills to ensure they facilitate positive interpersonal preceptorship experiences and create a positive clinical learning environment for their students. Furthermore, this programme will be available under creative comments and can be adapted for use by nurse educators internationally to suit local policies and procedures.

A significant strength of this study is the multiple methods design of the research, including qualitative interviews and a collaborative participatory design process. The project retained a user-centred design approach throughout, as their lived experience, expertise and knowledge of nursing preceptorship propelled the entire co-design and co-production of the new preceptorship education programme. Furthermore, the credibility and authenticity of the identified touchpoints were internally validated by the co design team. Although a diverse sample of SPP and professionals were recruited, most were female and of Irish nationality. An equal gender balance and representation of other diverse nationalities working and utilising general nursing hospitals in Ireland would provide a greater representation of cultural experiences and attitudes to nursing preceptorship relationships. However, hosting the one-to-one interviews and co-design workshops virtually facilitated broader demographic participation and increased transferability over a study carried out at a single hospital location. Overall, the co-design team actively engaged in the virtual co-design workshops. However, some voiced that they were uncomfortable with teleconferencing technology and preferred to engage over the phone. Bimonthly emails were therefore sent to all co-design members to keep the entire co-design team updated with all the latest outcomes of the workshops. Unfortunately, the researchers could not carry out observational studies in the hospital during the initial stages of the project due to Covid-19 restrictions. Direct observations could have provided further insight into behaviours and interactions during preceptorship. However, a comprehensive review of the literature identified and supported several key themes that emerged from the one-to-one interviews with each of the different cohorts suggesting that their experiences are universal and experienced internationally.

This is the first study to explore the EBCD of a new educational intervention. Replicating this study in other divisions of nursing education, such as care of older person education or surgical nursing education, would help to understand if the findings are generalisable and if EBCD is a suitable framework for co-design and co-development of other nursing education programmes and curricula. Furthermore, examining the co-designers’ experiences of partaking in this study could provide further insight into effective EBCD practices or limitations to creating a new educational programmes.

## Conclusion

This paper outlines an innovative adaption of the EBCD method to develop a new preceptorship education programme. EBCD is a valuable framework for developing human-centred educational resources that combine experiential knowledge (touchpoints) and scientific knowledge (literature-based knowledge). It facilitated the opportunity for all participants to have ample opportunities to suggest, create and refine ideas. It brought together individual viewpoints of all involved in nursing preceptorship to create a unique educational programme that captures real-life experiences. Combining interpersonal and communication skills theory with nursing theory, educational theory and behavioural change theory resulted in the development of what is hoped to be an authentic learner-centred educational programme that will equip preceptors with the knowledge and skills to effectively carry out their roles as a preceptor and build positive interpersonal relationships.

## Supplementary Information

**Additional file 1.** Semi structured Interview Guide.

**Additional file 2.** Student, Preceptor & Patient Touchpoints and overarching target behaviours to include in educational programme.

**Additional file 3.** GRIPP2 PPI reporting Checklist.

## Data Availability

The dataset(s) supporting the conclusions of this article are included within the article (and its additional file(s)).

## Abbreviations

EBCD: Experienced based co design
SPP: Students, preceptors, patients
CPC: Clinical placement coordinator
IP & C Skills: Interpersonal and communication skills
PPI: Public and patient involvement
