# Supplementary material for: Experienced based co design: nursing preceptorship educational programme
Source: Res Involv Engagem. 2022 Sep 17;8:53. doi: 10.1186/s40900-022-00385-3 (PMC9482750; doi:10.1186/s40900-022-00385-3)
Supplement: Supplementary file 1 — Additional file 1. Semi structured Interview Guide. [file 40900_2022_385_MOESM1_ESM.docx]

**Supplemental Information 1: Semi structured Interview Guide**

| Theme | Student | Preceptor | Patient |
| --- | --- | --- | --- |
| General Experiences | Can you tell me about your experiences of working with your preceptors on clinical placement ? What stage of your nursing programme? | Can you tell me about your experiences of being a nurse preceptor?  How long have you been a preceptor ? | Can you tell me in your own words how you came to be admitted to hospital and your subsequent experience of receiving care from nursing staff and student nurses?  Were you ever involved in a teaching session by a staff nurse for a student nurse? Can you tell me what that experience was like for you ? |
| Touch Points | Have there been any key moments that you feel have shaped your experiences of being involved in a nursing preceptorship either positively or negatively? | Have there been any key moments that you feel have shaped your experiences of being involved in a nursing preceptorship either positively or negatively? | Were there any key moments in this experience that stood out for you? Why?  What was that like for you ? |
| Suggestions for themes for the educational tool | The aim of this new educational package is to help educate trainee preceptors to create and facilitate positive interpersonal relationships. What key topics do you feel should be included in the new educational package to help achieve this? What would you want users of the package to learn? | The aim of this new educational package is to help educate trainee preceptors to create and facilitate positive interpersonal relationships what key topics do you feel should be included in the new educational package to help achieve this? What would you want users of the package to learn? | The aim of this new educational package is to help educate trainee preceptors to create and facilitate positive interpersonal relationships between the nurse, student, and patient. what key topics do you feel should be included in the new educational package to help achieve this? What would you want users of the package to learn? |
| Orientation | What have your experiences been to date with orientation to your placements by your preceptors? What was good , what was bad?  Do the preceptors include the patients in your orientation , do they explain the purpose of the professional relationship and your role to the patient ? Do you think it is important ? | As a preceptor when orientating a student to their placement , what do you feel are the key processes to facilitating a positive structured orientation experience? Do you include the patients in the student's orientation? Do you think it is important to include the patient at this stage?  What methods do you think are the most effective in orientating a student? | When you were in hospital did the staff nurse ever introduce themselves as the staff nurse and student and explain to you the relationship between them , and your involved in this relationship ?  How did you feel about being in this situation?  Was the purpose explained to you?  Do you think it is important the patient is involved in the student's orientation to their placement? |
| Individual Qualities of Preceptorship Members | What individual qualities do you think preceptors should exhibit to create positive relationships with students and patients?  What individual qualities do you think could negatively impact the relationships with students and patients? | What individual qualities do you think preceptors should exhibit to create positive relationships with students and patients?  What individual qualities do you think could negatively impact the relationships with students and patients? | What individual qualities do you think nurses/student nurses should exhibit to create positive relationships with students and patients?  What individual qualities do you think could negatively impact the relationships with students and patients? |
| Open Dialogue | Open dialogue has been established as a positive influencing factor in creating positive interpersonal relationships by creating an inviting and conducive learning environment; in other words, "keeping the channels of communication open" helps the relationships : from a student perspective how do you think a preceptor should achieve this with both the students and patients? | Open dialogue has been established as a positive influencing factor in creating positive interpersonal relationships by creating an inviting and conducive learning environment; in other words, "keeping the channels of communication open" helps the relationships . As a preceptor how do you think you can achieve this with both the students and patients? | What do you think are the best ways a nurse/student nurse can keep the channel of communication open with their patients ? |
| Feedback | Feedback is an important part of a nursing preceptorship; what has been your experience of preceptors' feedback to date?  In your opinion how do you think a preceptor can create a collaborative and bidirectional feedback process with students? Do you think the patient should be involved in the feedback process? | Feedback is an important part of a nursing preceptorship, as a preceptor how do you think preceptors can provide students with opportunities to actively partake in the process?  Do you include patients in the feedback process? If you were giving a student feedback in front of the patient, how do you think you can involve the patient in the process? How do you think that would made the patient feel?" "Would you ever give negative feedback in front of a patient to improve student performance? | A major role for the staff nurse is to provide the student feedback. Have you ever observed a nurse giving student feedback , how did you feel at the time, did the nurse involve you in the feedback process, would you like to be involved when the nurse is giving the student feedback? |
| Sense of Commitment | Did you feel your preceptor was committed to their role and educating you? What made you feel this?  What characteristics and qualities do you think a preceptor must demonstrate to students to show they are committed to their role of being your preceptor? | As a preceptor how do you show your students you are committed to your role of being their preceptor?  What characteristics and qualities do you think are fundamental to achieve this? | How do you feel a nurse/student nurse can demonstrate they are committed to your care and including you in this relationship? |
| Workplace Incivility | Unfortunately, workplace incivility such as rudeness, anger and humiliation has been reported by students while working with staff nurses ; did you ever observe or experience any incidences of this? If so, how did you feel at the time?  If answer is no: How do you think you would feel if you were the student or patient observing such behaviour? | Unfortunately, workplace incivility such as rudeness, anger and humiliation has been reported by students while in front of patients from preceptors; did you ever observe or experience any incidences of this? If so, how did you feel at the time?  If answer is no: How do you think you would feel if you were a student/patient? | Unfortunately, workplace incivility such as rudeness, anger and humiliation has been reported by students while working with staff nurses ; did you ever observe or experience any incidences of this? If so, how did you feel at the time?  If answer is no: How do you think you would feel if you were a patient observing such behaviour? |
| Any final thoughts or suggestions | Before we finish up is there anything else you would like to add, any final thoughts or suggestions you may have ? | Before we finish up is there anything else you would like to add, any final thoughts or suggestions you may have ? | Before we finish up is there anything else you would like to add, any final thoughts or suggestions you may have ? |
