# Supplementary material for: Experienced based co design: nursing preceptorship educational programme
Source: Res Involv Engagem. 2022 Sep 17;8:53. doi: 10.1186/s40900-022-00385-3 (PMC9482750; doi:10.1186/s40900-022-00385-3)
Supplement: Supplementary file 2 — Additional file 2. Student, Preceptor & Patient Touchpoints and overarching target behaviours to include in educational programme. [file 40900_2022_385_MOESM2_ESM.docx]

Supplemental Information 2: Student, Preceptor & Patient Touchpoints and overarching target behaviours to include in educational programme.

| Student Touchpoints | Examples | Overarching target behaviours to include in educational programme |
| --- | --- | --- |
| First Impressions | Positive Experience:  “It was noticeable when I got an excellent Preceptor because the first thing, I noticed was a warm welcome to the ward. That was unbelievable and simple things, a smile or “oh welcome” or “this is a student nurse who’s joining us”. Once you got that kind of initial feeling, you knew you had a good Preceptor. It was an excellent first impression.”  Negative Experience:  "Unfortunately, I've had many a Preceptor who just ignored the fact that I was even standing there. They didn't introduce you to anyone or didn't even say hello, which I found quite rude. There's just no need to be so unpleasant. You'd know straight away. Who would be the nurse that would help you because they'd offer you a seat for handover, a simple thing like that, rather than leaving you standing outside the group? The most negative experience for me was meeting with my preceptor. She was not prepared to meet me. She made me feel rejected and seemed to have so little interest in me when I spoke to her. That made me feel afraid, and I began to wonder how I would go back in there again tomorrow”. | Creating positive first impressions  Creating positive personal introductions  Creating welcoming environment |
| Teaching moments | Positive Experience:  "A nurse came to me and said, so, you're a second-year student. And she started asking me questions about the patients' conditions. It was a bit terrifying in the beginning. But after she finished, I was so thankful that she did that because she put me under that pressure, and I learned... If she asked me something and I said, "I don't know", she said, "That's fine, don’t worry about it. that's why I'm here to help you". Then she started explaining to me, and I was so grateful for that".  Negative Experience:  "Some preceptors, I've asked a question, and they get almost defensive of you asking the questions. It is like they are not sure of the answer. But then other preceptors will task you and admit they don't know the answer, and they'll ask you to research it, and you can come in the next day and teach them. They should not get offended or shut down the student for asking a question. It affects our learning". | Highlighting positive characteristics and barriers to effective teaching in the clinical environment and their impact on students learning.  Highlight impact levels of commitment on teaching have on student experience  Effective communication practises for teaching in the clinical environment. |
| Language for feedback | Positive Experience:  “When we left the room, my Preceptor gave me great affirmations about the whole thing, about how I spoke to the patient, that I had explained why and how I was doing the procedure. My Preceptor said, "OK, you did this brilliantly. You could have done this better for the next time. And she gave me clear things that she wanted to work on, and that was just so helpful and helped me learn even more".  Negative Experience:  “Any feedback I did receive without asking for it was usually: "you did this wrong without a full explanation on where I had gone wrong". So, it typically ends up with me apologising and saying I'll do it better next time. And that was the end of the feedback. It would be nice to receive positive verbal feedback "Well done, you've done very well". You want to hear that from time to time, not every day, but when you're doing something, you want to hear. Yes, you've done well. It gives you confidence, not just focusing on the negative”. | Effective and constructive language to use during feedback  Importance of regular verbal feedback  Appropriate locations to provide feedback |
| Feeling safe to learn | Positive Experience:  " I'll always remember when my preceptor said to me that "errors are normal, that making mistakes is human, and that perfection isn't expected of me. So don't be afraid to ask questions if you're not sure of anything. I would prefer you to ask them than pretend to know something". It made me feel comfortable, and I knew what was expected of me. I learned so much on that placement".  Negative Experience:  "I had already completed eight out of ten of my supervised medication rounds. And so, I was surprised when a preceptor I had never worked with before stood over me as I was getting the medication ready. Out of nowhere, she quite aggressively snapped the medication cup from my hand, saying, "you're taking too long; we will be here all day". I knew I was shaking, I was nervous, and it wasn't because I was scared to do this skill, but it was the intimidation that my preceptor put on me". | How to create a safe learning environment for students (psychological safety) |

| Preceptor Touchpoints | Examples | Overarching target behaviours to include in educational programme |
| --- | --- | --- |
| Feedback | Positive Experience:  “Getting good feedback from the students expressing their gratitude, saying, "thank you, thanks for all your help today I really learned a lot". Or "you helped me to understand that medical condition", or "you showed me how to do that procedure, thank you". Those are positive experiences. I do enjoy teaching. I like imparting my knowledge to the student nurses. They are the next generation of nurses".  Providing feedback:  “If there are issues or areas that the student needs to improve on, you must say it to the student but do it privately. I would never really give negative feedback in front of anybody, even if it's something minimal. We've all been the student going into meetings, they are so nervous. I would never want somebody to feel down over getting negative feedback in front of anybody. You don't want to make them to feel like they're no good. For your colleagues or patients to see and hear you giving negative feedback would make it so much worse for the student". | Creating bidirectional feedback opportunities for students and preceptors  Time and locations to provide feedback |
| Pressure and guilt in failing a student | “I have had instances where I have had to fail students, which has been upsetting for both the student and me. You can sort of feel; I suppose maybe are you failing them as opposed to them failing you! The spotlight is on you a lot of the time. It can be time-consuming; you put a learning plan in place, put a lot of work into it, and do your best to ensure that somebody is competent. But at the end of the day, you still have to say it's a health care profession and specific standards you need to achieve. It's a massive pressure on me to say, no, you're not competent. There's a bit of guilt that you're failing them; they're under your wing. I'd feel anxious if I had passed the person, you know, am I sending them out to be somebody's nurse”. | Strategies to deal with pressure or feelings of guilt if failing a student i.e. How to provide effective constructive feedback. |
| Creating a safe learning space for students | "When talking to students, you should make them comfortable and at ease. They normally do good then, and you give them good feedback. You see them walking out with a smile. They're always very thankful. And you get the sense that they're excited to come back. You might get some good feedback from students that they have felt you have helped them learn or helped them enjoy the day or whatever it may be. It's making them comfortable chatting to you and their patients. It's rewarding".  “As a staff nurse, there is a lot you don’t know, but the students expect you to know. You don’t want them to think you are thick. That's why you tell them to look it up. If I had the time, id would say come on, and we can look it up. And then we'll both know, instead I send them off by themselves"  "There can be a culture in my ward. The (students) are scared to ask questions, they don't want to annoy the nurse, so they keep away from them. Then at the student's meeting, the preceptors give the students negative feedback. The preceptors feel like the students aren't interested and don't want to be there. That's what kind of adds fuel to the fire then for them. Then the students are like; I can't be right either way. Do you want me to ask questions? Do you not want me to ask questions? It's challenging for students to learn new nursing skills and figure out what each nurse is like? Personalities are very different for different people, and different nurses will expect you to do different things. So, it's tough for the students ". | Fostering safe learning environments for students  Open communication concerning lack of knowledge; create a psychological safe learning environment  How to create an open and collaborative relationship where student/preceptor set expected behaviours in the relationship where students can feel safe to ask questions |
| Socio cultural factors | "I've seen a staff member speak inappropriately to a student in front of other staff. The nurse said to the student, "why are you giving your opinion on the situation" referring to a patients nursing care management, "no one asked you, go back to the nurses' station and finish the notes. The student was blatantly excluded from the conversation. It's hard to watch because it is a cultural thing on the ward, and you don't want to say anything in case they start speaking to you like that then too". | Highlighting the impact workplace incivility has on students and colleagues in psychological safety |
| Environmental Factors: Time | "The ward might be too busy and short staffed, and you can't give your time to them (student). Fourth years, you know, they're well able to do their own thing and come to you if they need to, but especially for first years, you feel like you can't give them the time they deserve or need to learn. It makes you feel a bit crap that you can't give them the time. They might go home at the end of the day and say either I did nothing for the day because I was standing around or my Preceptor had no interest in me. I don’t like the feeling that they think you don't want to look after them. It can be frustrating". | Open communication techniques to inform students of time constraints and difficulties to create a greater understanding of preceptor’s perspective and position |

| Patient Touchpoints | Examples | Overarching target behaviours to include in educational programme |
| --- | --- | --- |
| Positive interaction with students | "My interaction with the student nurses and what I've seen around me are very good at interacting with the patient. And I mean, we used to have some fun up there, and they were so good, nothing was too much trouble for them if you know what I mean...... And months afterwards, when I went back up for a Check-Up, some nurses said, "l saw you there, so I thought I'd come over to see how you're getting on". I thought, god, so many people coming in, they're not going to recognise anybody. But they came. They saw you going in. How are you? Which I thought was nice. If I had to go back into the hospital, I thought, thank god, that nice girl would be here". | Making the patient feel welcome |
| Patients involved in a teaching session | "I think it's really bad when the nurse asks you whether the student can observe when they are already in the room. You don't have a choice then as you don't want to come across as being difficult or making them (student) feeling unwanted, or you feel the nurse may not spend as much time with you then”  "I want to be involved in giving feedback. For example, when I got my dressing changed, the pain I experienced was very subjective. The nurse who has been putting on the bandage was clearly used to doing it a particular way for years. She never asked me how I liked it to be changed or how my pain was. So, I think it necessary to get patient feedback. It's good for students to hear the patients' experiences and learn from them. But I would like to give feedback to the student directly after the nurse has gone so that the student knows next time. I would not want to make it a negative experience by putting the student on the spot and saying it in front of the nurse. I'd give it in a roundabout way rather than directly pointing it out as I know they're learning". | Guidelines on how to approach patients to seek permission for students to observe or be involved in the patient’s care as a teaching session and involve patient in the feedback process. |
| Being treated like a human and not a patient | "She (the student) walks into the room and says, good morning, everybody. How are you this morning? Everybody said, (student name), how are you? And she's like, oh, God, I'm so tired. I stayed up half the night watching a film instead of doing my homework. You know that carry-on, and everyone is just starting to smile and laugh and, you know, I must admit, genuinely, they were the nicest bunch of nurses I've ever come across in my life. Just normal people who treated us like humans and not a patient". | Importance of person-centred communication i.e., communication beyond patients’ care. |
| Continued communication of patients’ care. | “ So, I’ve had really good experiences in hospital. All the nurses and doctors seem to coordinate and communicate with the patients. You get a sense of being looked after. I felt like my issue was being dealt with, and I had confidence in the care I was receiving. The culture is there. They look after the patient and work together as a team, communicate with you and are endearing towards you. Asking if you are, okay? Or if they were going home for the day, telling you, so you know what’s going on”. | Importance of open and continued communication with patients on their care and daily routine with their nurse/student to make them feel part of the team. |
