# Supplementary material for: Experienced based co design: nursing preceptorship educational programme
Source: Res Involv Engagem. 2022 Sep 17;8:53. doi: 10.1186/s40900-022-00385-3 (PMC9482750; doi:10.1186/s40900-022-00385-3)
Supplement: Supplementary file 3 — Additional file 3. GRIPP2 PPI reporting Checklist. [file 40900_2022_385_MOESM3_ESM.docx]

**Supplemental Information 3: GRIPP2 PPI reporting Checklist**

| **Section 1: Abstract of paper** | Description | Reported on Page |
| --- | --- | --- |
| 1a: Aim | Report the aim of the study | 1 |
| 1b: Methods | Describe the methods used by which patients and the public were involved | 1 |
| 1c: Results | Report the impacts and outcomes of PPI in the study | 1 |
| 1d:Conclusions | Summarise the main conclusions of the study | 2 |
| 1e: Keywords | Keywords Include PPI, "patient and public involvement," or alternative terms as keywords | 2 |
| **Section 2: Background to paper** |  |  |
| 2a: Definition | Report the definition of PPI used in the study and how it links to comparable studies | 4 |
| 2b: Theoretical underpinnings | Report the theoretical rationale and any theoretical influences relating to PPI in the study | 3-5 |
| 2c: Concepts and theory development | Report any conceptual or theoretical models, or influences, used in the study | 5-6 |
| **Section 3: Aims of paper** |  |  |
| Aim | Report the aim of the study | 5 |
| **Section 4: Methods of paper** |  |  |
| 4a: Design | Provide a clear description of methods by which patients and the public were involved | 5-17 |
| 4b: People involved | Provide a description of patients, carers, and the public involved with the PPI activity in the stud | 6-7 |
| 4c: Stages of involvement | Report on how PPI is used at different stages of the study | 5-17 |
| 4d: Level or nature of involvement | Report the level or nature of PPI used at various stages of the study | 5-17 |
| **Section 5: Capture or measurement of PPI impact** |  |  |
| 5a: Qualitative evidence of impact If applicable | report the methods used to quantitatively measure or assess the impact of PPI | n/a |
| 5c: Robustness of measure If applicable | report the rigour of the method used to capture or measure the impact of PPI | n/a |
| Section 6: Economic assessment | Economic assessment If applicable, report the method used for an economic assessment of PPI | n/a |
| Section 7: Study results |  |  |
| 7a: Outcomes of PPI | Report the results of PPI in the study, including both positive and negative outcomes | 12-17 |
| 7b: Impacts of PPI | Report the positive and negative impacts that PPI has had on the research, the individuals involved (including patients and researchers), and wider impacts | 17 |
| 7c: Context of PPI | Report the influence of any contextual factors that enabled or hindered the process or impact of PPI | 18-19 |
| 7d: Process of PPI | Report the influence of any process factors, that enabled or hindered the impact of PPI | 19 |
| 7ei: Theory development | Theory development Report any conceptual or theoretical development in PPI that have emerged | 20 |
|  | development Report evaluation of theoretical models, if any | n/a |
| **Section 8: Discussion and conclusions** |  |  |
| 8a: Outcomes | Comment on how PPI influenced the study overall. Describe positive and negative effects | 12-17 |
| 8b: Impacts | Comment on the different impacts of PPI identified in this study and how they contribute to new knowledge | 12-17 |
| 8c: Definition | Comment on the definition of PPI used (reported in the Background section) and whether or not you would suggest any changes | 19 |
| 8d: Theoretical underpinnings | Comment on any way your study adds to the theoretical development of PPI | 19 |
| 8e: Context | Comment on how context factors influenced PPI in the study 8f: Process Comment on how process factors influenced PPI in the study | 19 |
| 8f: Process | Comment on how process factors influenced PPI in the study | 19 |
| 8 g: Measurement and capture of PPI impact If applicable | comment on how well PPI impact was evaluated or measured in the study | n/a |
| 8 h: Economic assessment If applicable | discuss any aspects of the economic cost or benefit of PPI, particularly any suggestions for future economic modelling | n/a |
| 8i: Reflections/critical perspective | Comment critically on the study, reflecting on the things that went well or not so well | 19 |
